# A New Dioic Acid from a *wbl* Gene Mutant of Deepsea-Derived *Streptomyces somaliensis* SCSIO ZH66

**DOI:** 10.3390/md14100184

**Published:** 2016-10-17

**Authors:** Huiming Huang, Huayue Li, Yanhong Qiu, Lukuan Hou, Jianhua Ju, Wenli Li

**Affiliations:** 1 Key Laboratory of Marine Drugs, Ministry of Education, School of Medicine and Pharmacy, Ocean University of China, Qingdao 266003, China; hmhuang1988@163.com (H.H.); lihuayue@ouc.edu.cn (H.L.); qq2yanhong@163.com (Y.Q.); houlukuan1991@163.com (L.H.); 2 CAS Key Laboratory of Marine Bio-resources Sustainable Utilization, Guangdong Key Laboratory of Marine Materia Medica, RNAM Center for Marine Microbiology, South China Sea Institute of Oceanology, Chinese Academy of Sciences, 164 West Xingang Road, Guangzhou 510301, China; jju@scsio.ac.cn; 3 Laboratory for Marine Drugs and Bioproducts of Qingdao National Laboratory for Marine Science and Technology, Qingdao 266237, China

**Keywords:** deepsea-derived *Streptomyces*, dioic acid, *wblA_so_*

## Abstract

The *wblA_so_* gene functions as a global regulatory gene in a negative manner in deepsea-derived *Streptomyces somaliensis* SCSIO ZH66. A new dioic acid (**1**) as well as two known butenolides (**2** and **3**) were isolated from the *ΔwblA_so_* mutant strain of *S. somaliensis* SCSIO ZH66. The structure of **1** was elucidated by a combination of spectroscopic analyses, including MS and NMR techniques. In the cell growth inhibitory evaluation, compound **3** exhibited moderate activity against the human hepatic carcinoma cell line (Huh7.5) with an IC_50_ value of 19.4 μg/mL, while compounds **1** and **2** showed null activity up to 100 μg/mL.

## 1. Introduction

Marine-derived bioactive compounds and their novel chemical scaffolds have been shown to be attractive starting points for drug discovery programs [1,2]. Over the last few years, the actinomycetes isolated from marine environments have attracted considerable attention, because of their great potential for producing a large diversity of bioactive compounds [3,4]. However, discovery of structurally novel secondary metabolites from microbes is becoming more and more difficult, as the rate of rediscovery of known compounds is increasing [5,6]. Recent genome sequencing revealed that actinomycetes can genetically synthesize secondary metabolites beyond those that were isolated under standard cultivation conditions [7,8,9]. However, a number of gene clusters in actinomycetes are silent or only low-expressed and thus are defined as cryptic or orphan clusters [10].

**Figure 1:**
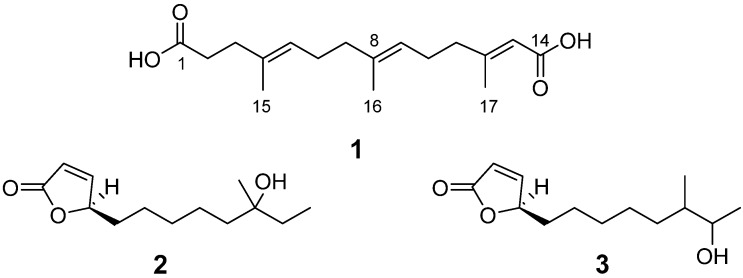
Structures of compounds **1**–**3**.

Manipulation of global regulators is an effective strategy for the activation of cryptic gene clusters [11]. In the previous study, we obtained a series of anti-MRSA (methicillin-resistant *Staphylococcus aureus*) α-pyrone compounds (violapyrones A–C, H, and J) from the deepsea-derived *S. somaliensis* SCSIO ZH66 by disruption of the negative global regulatory gene *wblA_so_* [12]. In our continuous search for significantly enhanced compounds in the *ΔwblA_so_* mutant strain, one new dioic acid (**1**) together with two known butenolides (**2** and **3**) were isolated (Figure 1). We describe herein the isolation, structure elucidation, and biological evaluation of these accumulated compounds.

## 2. Results and Discussion

**Figure 2:**
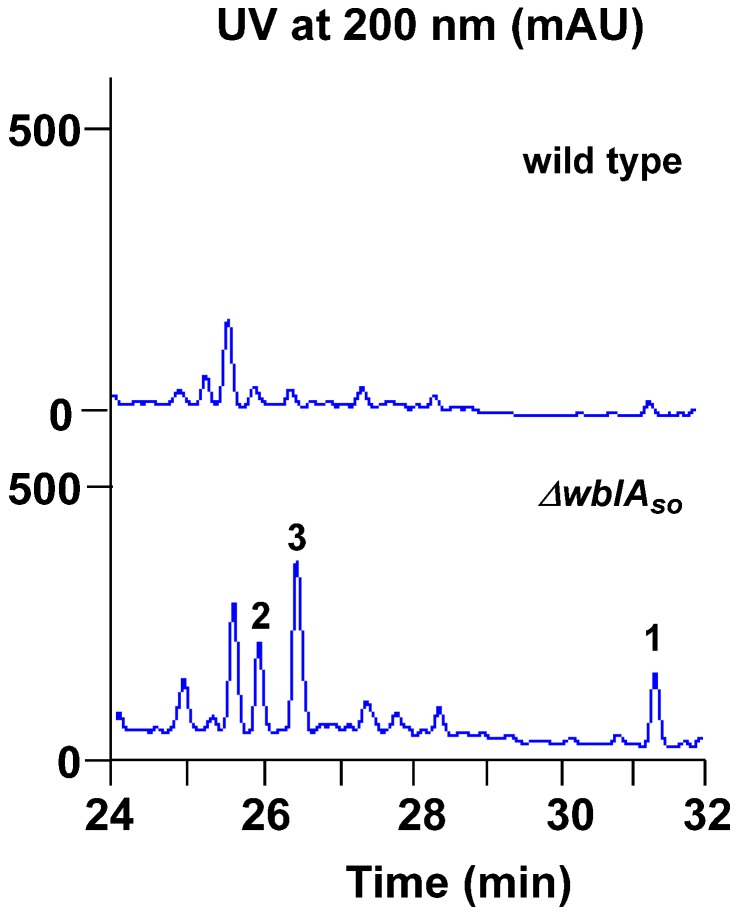
Comparative HPLC analysis of the secondary metabolites in the culture extracts of wild-type *S. somaliensis* SCSIO ZH66 and its *ΔwblA_so_* mutant strain. The notably enhanced peaks (**1**–**3**) in the *ΔwblA_so_* mutant strain were numbered.

The *ΔwblA_so_* mutant of *S. somaliensis* SCSIO ZH66 was constructed in our previous study [12]. The fermentation broths of the wild-type and the *ΔwblA_so_* mutant strains were extracted with EtOAc, respectively, and were subsequently subjected to high-performance liquid chromatography (HPLC) analysis (Figure 2), in which we observed three significantly enhanced peaks (**1**–**3**) in the *ΔwblA_so_* mutant compared with those in the wild-type strain at wavelengths of 200 nm. With the massive fermentation of the *ΔwblA_so_* mutant, compounds **1**–**3** were isolated and further identified via NMR assignments.

**Figure 3:**
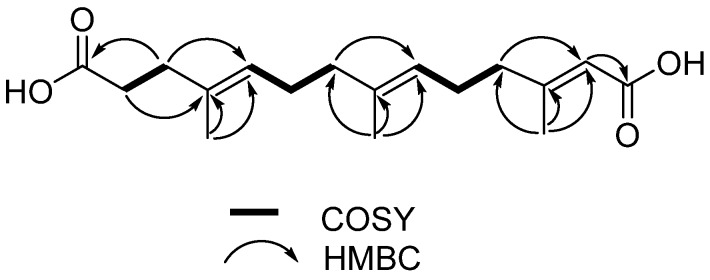
Key ^1^H–^1^H COSY and HMBC correlations of compound **1**.

**Figure 4:**
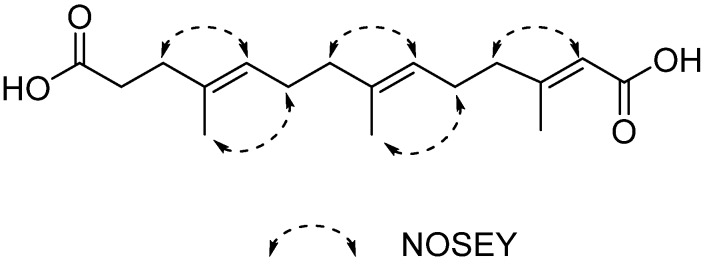
Key NOSEY correlations of compound **1**.

Compound **1** was obtained as a white, amorphous solid. The molecular formula of **1** was established as C_17_H_26_O_4_ on the basis of HR-ESIMS data ([M + Na]^+^ at *m/z* 317.1723) (Figure S1). The structure of **1** was determined by 1D (^1^H, ^13^C) and 2D NMR (COSY, HSQC and HMBC) data (Table 1). The ^1^H-NMR spectrum of **1** disclosed three methyls (δ_H_ 1.55, 1.56, 2.07) and six methylenes (δ_H_ 2.26, 2.16, 2.02, 1.93, 2.12, 2.12), and the ^13^C-NMR spectrum showed 17 carbon signals. The ^1^H–^1^H COSY spectrum established the spin systems of H-2 (δ_H_ 2.26)/H-3 (δ_H_ 2.16), H-5 (δ_H_ 5.09)/H-6 (δ_H_ 2.02)/H-7 (δ_H_ 1.93) and H-9 (δ_H_ 5.07)/H-10 (δ_H_ 2.12)/H-11 (δ_H_ 2.12) (Figure 3); the HMBC correlations from H-3 to C-1 (δ_C_ 174.1) and C-5 (δ_C_ 124.0), from H-7 to C-9 (δ_C_ 123.1), from H-11 to C-13 (δ_C_ 116.2), and from H-13 (δ_H_ 5.58) to C-14 (δ_C_ 167.4) established the main aliphatic chain of **1**. The HMBC correlations from the methyl protons H-15 (δ_H_ 1.55) to C-4 (δ_C_ 133.6), from H-16 (δ_H_ 1.56) to C-8 (δ_C_ 135.2), and from H-17 (δ_H_ 2.07) to C-12 (δ_C_ 158.4) confirmed the location of three methyl groups (Figure 3). Moreover, the configurations of three double bonds were confirmed to be *trans* by NOSEY correlations of H-3/H-5, H-7/H-9, H-11/H-13, H-15/H-6, and H-16/H-10 (Figure 4). The ^13^C chemical shifts of C-1 and C-14, together with HR-ESIMS data, revealed two carboxyl groups in **1**. Thus, the structure of **1** was finally identified as 3,7,11-trimethyl-2,6,10-triene-1,14-tetradecyl dioic acid.

Compound **2** was isolated as a light yellow oil. The molecular formula of **2** was established as C_13_H_22_O_3_ on the basis of HR-ESIMS data ([M + Na]^+^ at *m/z* 249.1337) (Figure S2). Compound **2** was identified as (4*S*)-4,10-dihydroxy-10-methyl-dodec-2-en-1,4-olide by comparison of ^1^H and ^13^C-NMR data (Table S1) with those reported in [13].

Compound **3** was isolated as a light yellow oil. The molecular formula of **3** was established as C_13_H_22_O_3_ on the basis of HR-ESIMS data ([M + Na]^+^ at *m/z* 249.1336) (Figure S3). Compound **3** was identified as (4*S*)-4,11-dihydroxy-10-methyl-dodec-2-en-1,4-olide by comparison of ^1^H and ^13^C-NMR data (Table S1) with those reported in [13].

Butenolides are a family of α,β-unsaturated lactones, and their saturated analogs act as signaling substances in bacteria to enhance sporulation or induce metabolite production [14]. Some butenolides have been reported to show cytotoxicity [13] or antimicrobial activities [15,16]. In the evaluation for cell growth inhibitory effects, compound **3** exhibited moderate growth inhibition against the human hepatic carcinoma cell line (Huh7.5) with an IC_50_ value of 19.4 μg/mL, while compounds **1** and **2** showed null activity up to 100 μg/mL. In the test of antimicrobial activity, none of these compounds showed significant activity against multi-drug resistant strains (*Staphylococcus aureus* CCARM 3090, *Escherichia coli* CCARM 1009, *Enterococcus faecalis* CCARM 5172, *Enterococcus faecium* CCARM 5203, and *Salmonella typhimurium* CCARM 8250).

## 3. Experimental Section

### 3.1. General Experimental Procedures

HPLC was performed on an Agilent 1260 Infinity equipment with Diode Array Detector (DAD) (Agilent, Waldbronn, Germany). NMR spectra were recorded on Agilent DD2-500 spectrometers. Chemical shifts were reported with reference to the respective solvent peaks and residual solvent peaks (δ_H_ 2.50 and δ_C_ 39.5 ppm for DMSO-*d*_6_). HR-ESIMS data were obtained on a Q-TOF Ultima Global GAA076 LC-MS spectrometer (Waters Corporation, Milford, MA, USA).

### 3.2. Strains and Culture Conditions

The *S. somaliensis* SCSIO ZH66 (CGMCC NO. 9492) was isolated from the deep-sea sediment collected at a depth of 3536 m in the South China Sea (120°0.250′ E; 20°22.971′ N) [17]. The *ΔwblA_so_* mutant was obtained as described previously [12]. The strains were grown at 30 °C on a MS (mannitol-soy flour) medium for sporulation.

### 3.3. Fermentation, Extraction, and Isolation of the Compounds

For each fermentation, strain spores were inoculated into 200 mL of fermentation medium (1% soluble starch, 2% glucose, 4% corn syrup, 1% yeast extract, 0.3% beef extract, 0.05% MgSO_4_·7H_2_O, 0.05% KH_2_PO_4_, 0.2% CaCO_3_, and 3% sea salt, pH = 7.0), which was further supplemented with 1.5% XAD-16 resin when fermenting the *ΔwblA_so_* mutant, and incubated at 30 °C, 220 rpm for 7 days. Analytical HPLC was performed on an Eclipse C18 column (5 μm, 4.6 × 150 mm) developed with a linear gradient from 20% to 70% B/A in 40 min (phase A: 0.1% formic acid in H_2_O; phase B: 100% acetonitrile supplemented with 0.1% formic acid). A total of 20 L of culture was made by this method. The fermentation cultures were harvested via centrifugation, and the supernatant was extracted twice with an equal volume of ethyl acetate. The combined ethyl acetate extracts were concentrated in vacuo to afford residue A. The precipitated mycelia and XAD-16 resin were extracted twice with acetone. The extracts were combined, and acetone was evaporated in vacuo to yield residue B. Both residues from fermentation broths and mycelia cakes were combined to afford crude extract (6.0 g). They were applied to reversed-phase C18 open column, eluting with a gradient eluent of 20%–100% methanol to give five fractions (Fr.1~Fr.5). Compound **1** (3.8 mg) was obtained by further separation of Fr.4 eluting with gradient solvents (phase A: 0.1% formic acid in H_2_O; phase B: 100% acetonitrile supplemented with 0.1% formic acid, 2 mL/min, UV detection at 200 nm) using a semi-preparative HPLC column (YMC-Pack ODS-AA C18 column, 120 Å, 250 × 10 mm, 5 μm). Fr.3 was also further subjected to semi-preparative HPLC eluting with gradient solvents to afford compounds **2** (2.9 mg) and **3** (5.5 mg).

### 3.4. Biological Assays

Viabilities of human hepatic carcinoma cell line (Huh7.5) were measured with a MTT assay. Briefly, logarithmically growing cells were trypsinized from culture dishes and placed into 96-well plate and cultured at 37 °C for 24 h. Cells were treated with the varying concentrations of each compound, and then 20 μL of a MTT solution (5 mg/mL) were added to each well. After incubating for 4 h, the medium was removed, and 150 μL of DMSO were added to each well to dissolve purple crystals of formazan via shaking at 260 rpm for 10 min. Absorbance was measured at 490 nm by a multi-detection microplate reader (infinite M1000 Pro, TECAN, Mannedorf, Switzerland). The 50% inhibitory concentration (IC_50_) value was determined as the concentration that caused 50% inhibition of cell proliferation [18,19].

The antibacterial activity against five multi-drug resistant (MDR) strains (*S. aureus* CCARM 3090, *E. coli* CCARM 1009, *E. faecalis* CCARM 5172, *E. faecium* CCARM 5203, and *S. typhimurium* CCARM 8250) was tested by the radial diffusion assay as previously described [12].

## 4. Conclusions

A new dioic acid (**1**) and two known butenolides (**2** and **3**) were isolated from the *ΔwblA_so_* mutant strain of deepsea-derived *S. somaliensis* SCSIO ZH66. In the evaluation for cell growth inhibitory effects, compound **3** showed moderate activity against the human hepatic carcinoma cell line (Huh7.5) with an IC_50_ value of 19.4 μg/mL. Isolation of a novel dioic acid (**1**) in this study indicated that the manipulation of global regulators can be used as an effective method for the accumulation of novel secondary metabolites.

## Figures and Tables

**Table 1:** ^1^H and ^13^C-NMR data of compound **1** in DMSO-*d*_6_ (δ in ppm, *J* in Hz).

Position	δ_H_	δ_C_
1	-	174.1
2	2.26 (2H, m)	32.7
3	2.16 (2H, t, 8.4)	34.2
4	-	133.6
5	5.09 (1H, m)	124.0
6	2.02 (2H, m)	26.1
7	1.93 (2H, t, 7.8)	39.1
8	-	135.2
9	5.07 (1H, m)	123.1
10	2.12 (2H, m)	25.5
11	2.12 (2H, m)	40.0
12		158.4
13	5.58 (1H, s)	116.2
14		167.4
15	1.55 (3H, s)	15.8
16	1.56 (3H, s)	15.8
17	2.07 (3H, s)	18.2

## Supplementary Materials

The following are available online at http://www.mdpi.com/1660-3397/14/10/184/s1. Figure S1: Spectral data of compound **1**; Figure S2: Spectral data of compound **2**; Figure S3: Spectral data of compound **3**; Table S1: ^1^H and ^13^C-NMR Data of compound **2** and **3**.
