# Supplementary material for: A New Dioic Acid from a wbl Gene Mutant of Deepsea-Derived Streptomyces somaliensis SCSIO ZH66
Source: Mar Drugs. 2016 Oct 17;14(10):184. doi: 10.3390/md14100184 (PMC5082332; doi:10.3390/md14100184)
Supplement: Supplementary file 1 [file marinedrugs-14-00184-s001.pdf]

Supplementary Materials: A New Dioic Acid from a *wbl* Gene Mutant of Deepsea-Derived *Streptomyces somaliensis* SCSIO ZH66

Huiming Huang, Huayue Li, Yanhong Qiu, Lukuan Hou, Jianhua Ju and Wenli Li

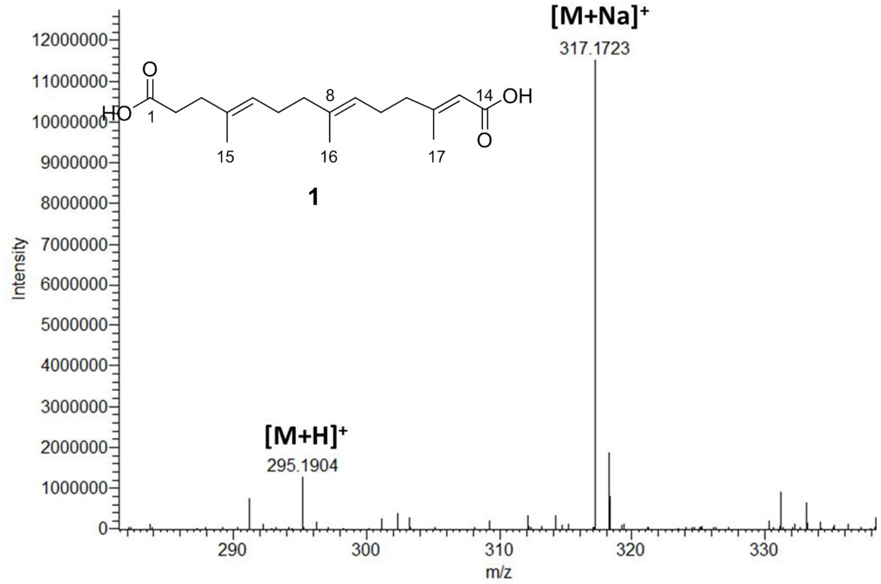

(**A**)

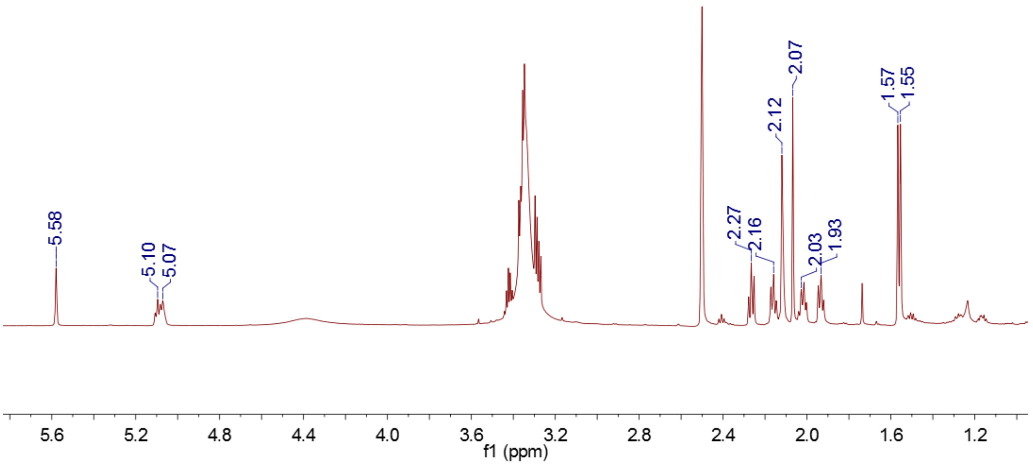

(**B**)

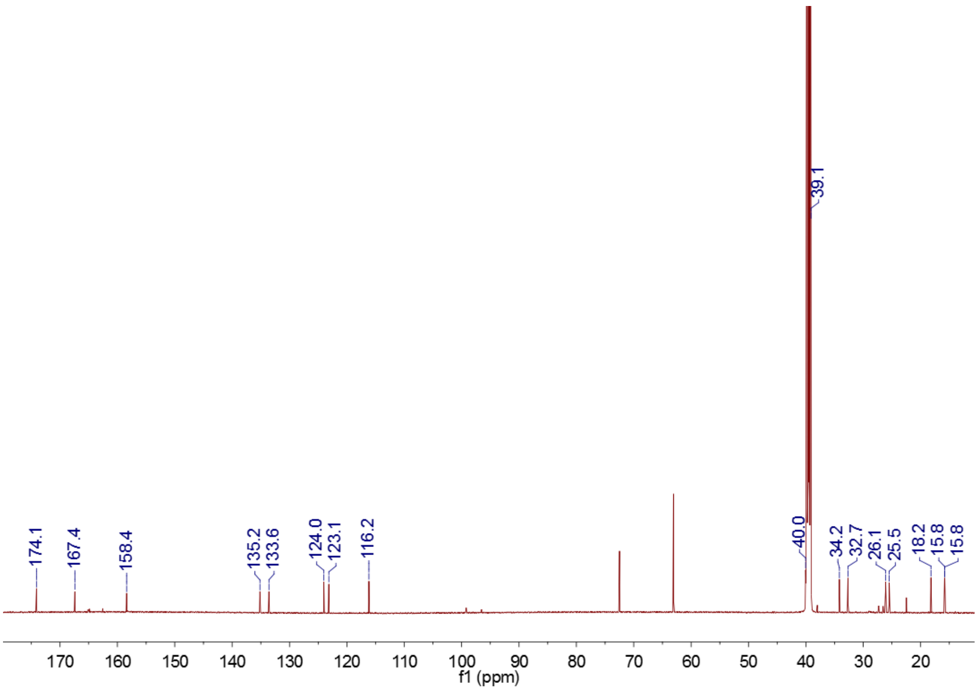
174.1
167.4
158.4
135.2
133.6
124.0
123.1
116.2
39.1
40.0
34.2
32.7
26.1
25.5
18.2
15.8
15.8
170
160
150
140
130
120
110
100
90
80
70
60
50
40
30
20
f1 (ppm)

(**C**)

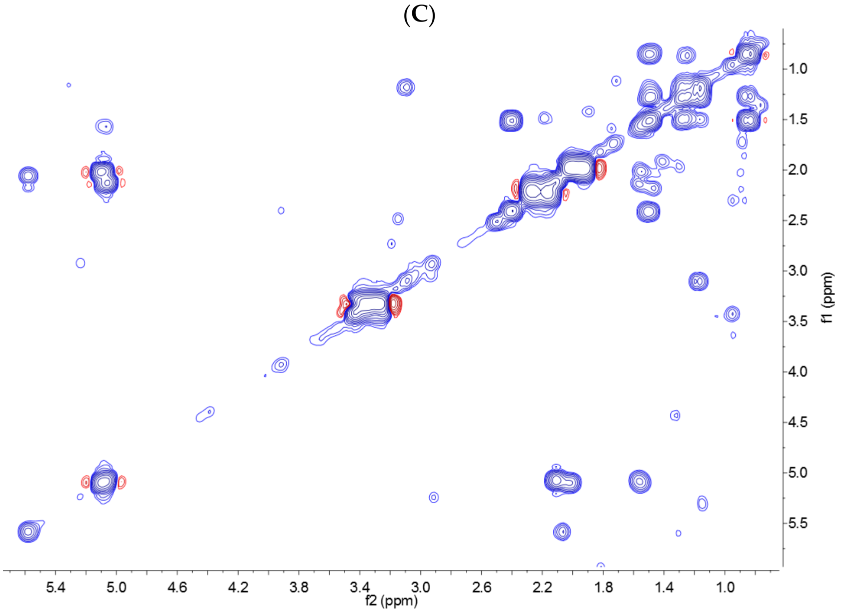
5.4
5.0
4.6
4.2
3.8
3.4
3.0
2.6
2.2
1.8
1.4
1.0
f2 (ppm)
1.0
1.5
2.0
2.5
3.0
3.5
4.0
4.5
5.0
5.5
f1 (ppm)

(**D**)

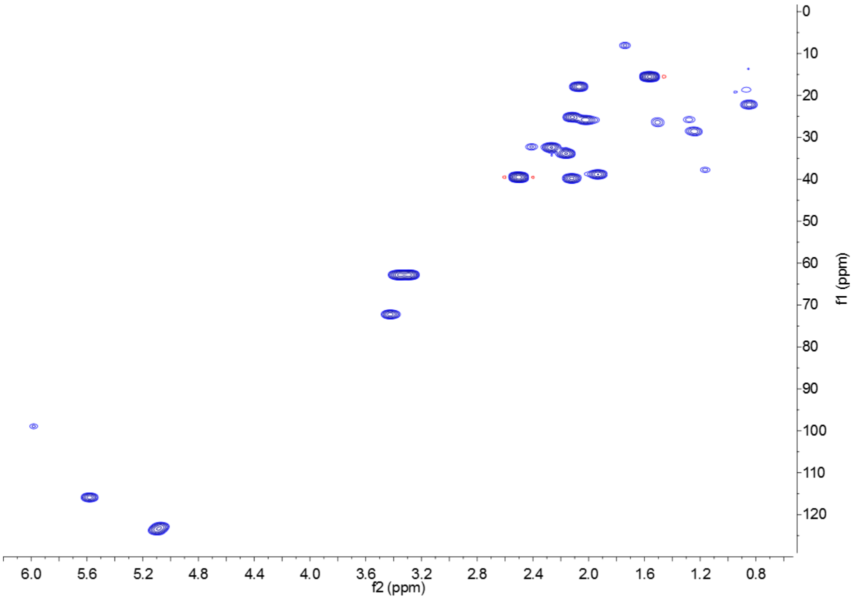

(**E**)

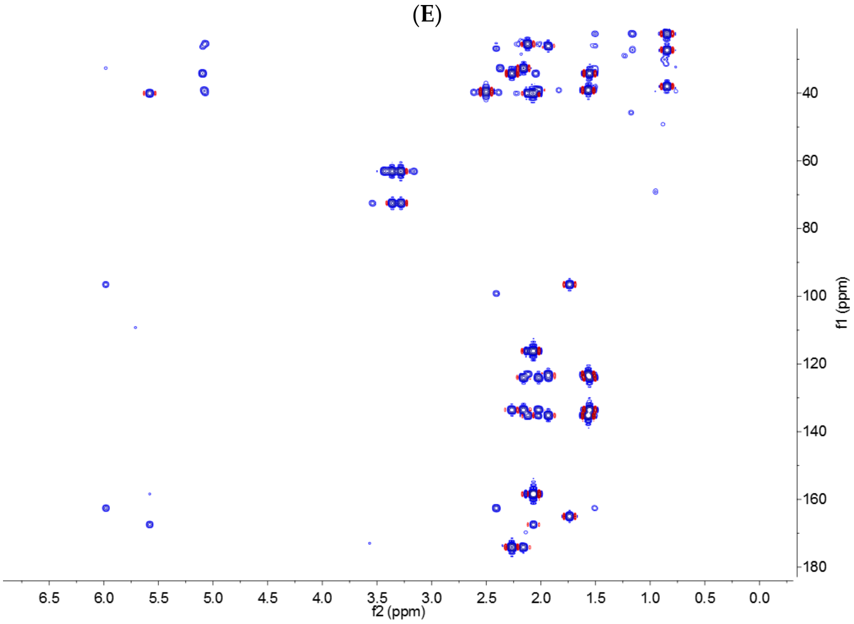

(**F**)

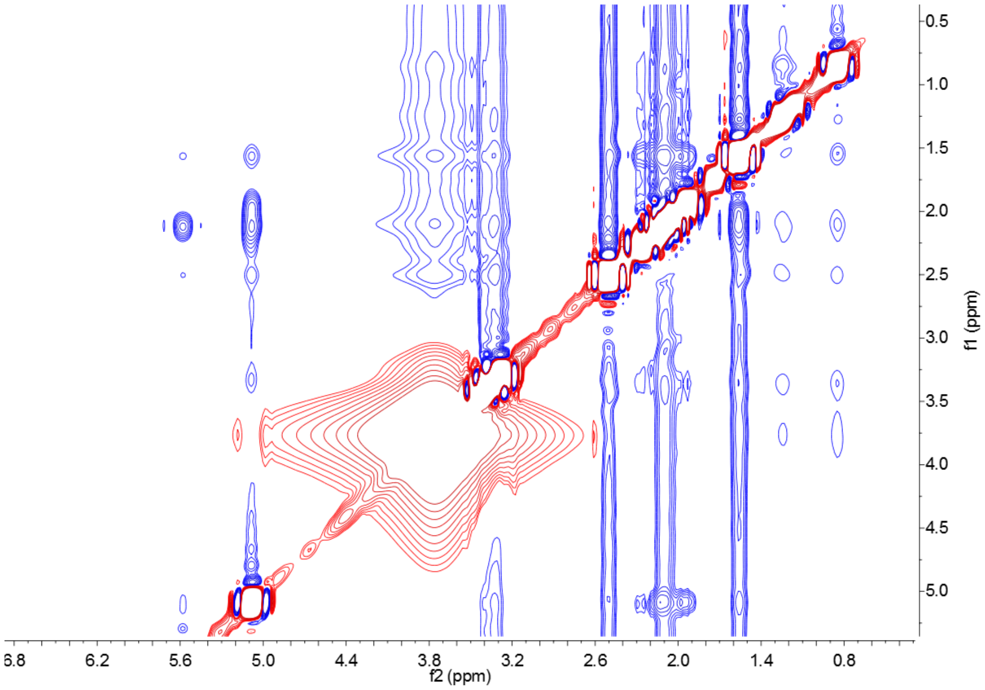

(**G**)

Figure S1. Spectral data of compound **1**; (**A**) The HR-ESIMS spectrum of compound **1**; (**B**) The ^{1}H-NMR spectrum of compound **1** in DMSO-*d*6; (**C**) The ^{13}C-NMR spectrum of compound **1** in DMSO-*d*6; (**D**) The ^{1}H-^{1}H COSY spectrum of compound **1** in DMSO-*d*6; (**E**) The HSQC spectrum of compound **1** in DMSO-*d*6; (**F**) The HMBC spectrum of compound **1** in DMSO-*d*6; (**G**) The NOESY spectrum of compound **1** in DMSO-*d*6.

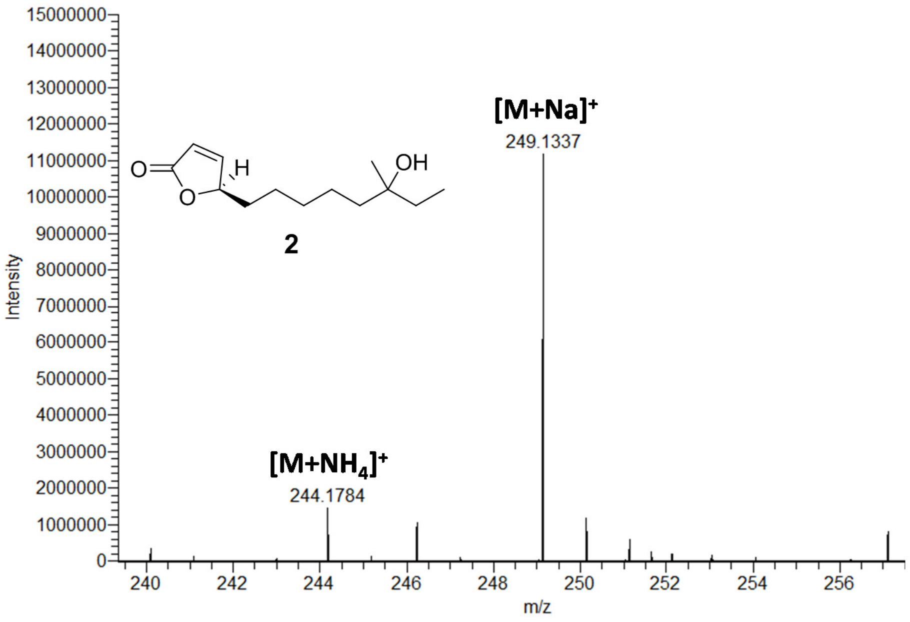

(**A**)

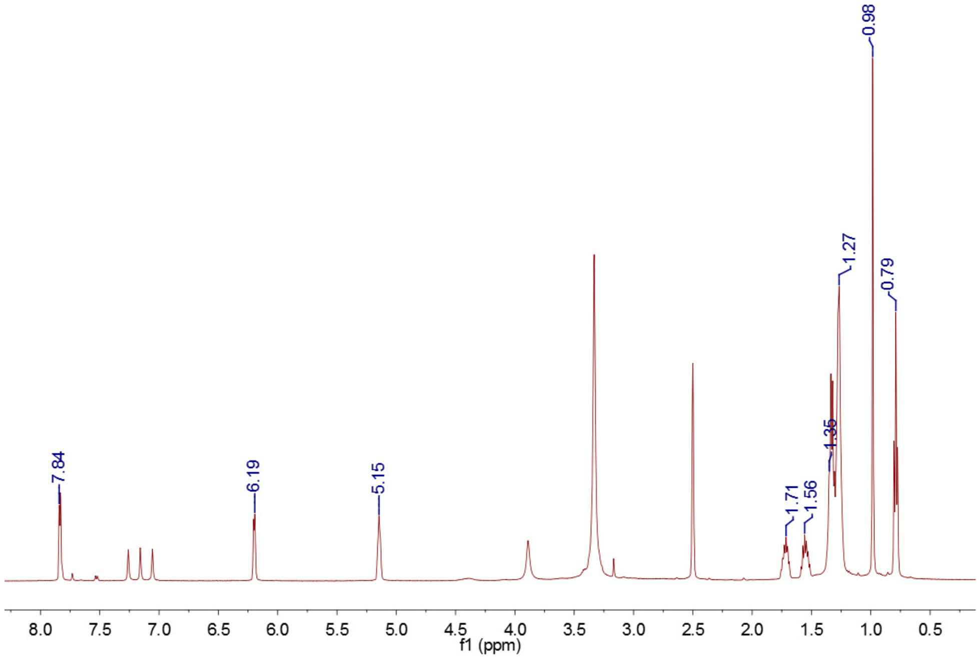

(**B**)

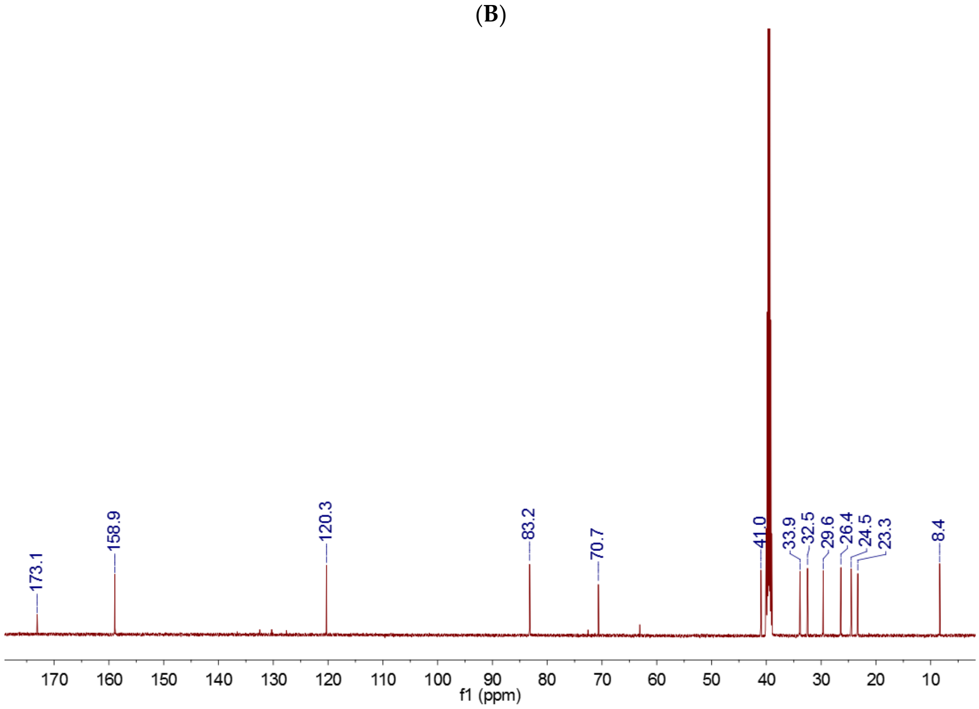

(**C**)

Figure S2. Spectral data of compound **2**; (**A**) The HR-ESIMS spectrum of compound **2**; (**B**) The ^{1}H-NMR spectrum of compound **2** in DMSO-d_6; (**C**) The ^{13}C-NMR spectrum of compound **2** in DMSO-d_6.

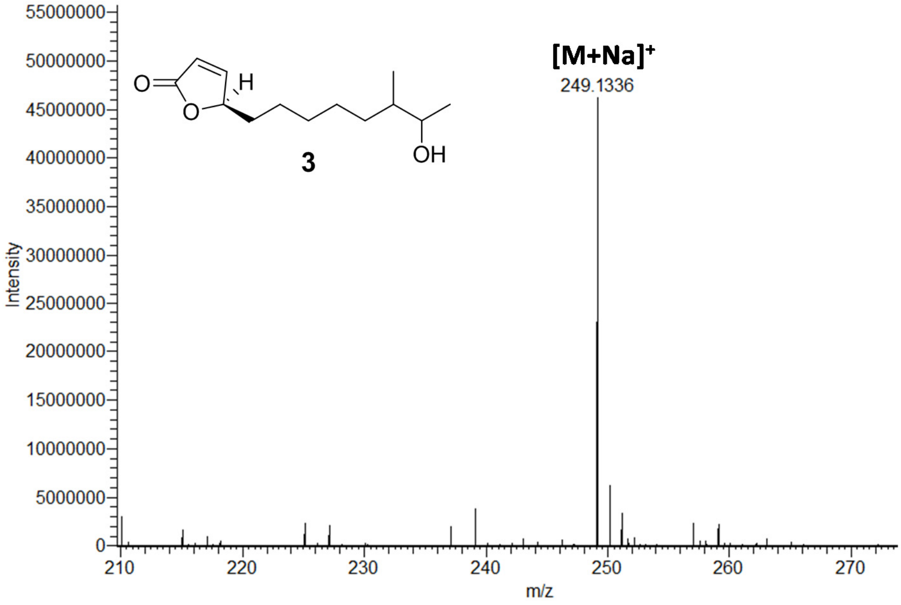

(**A**)

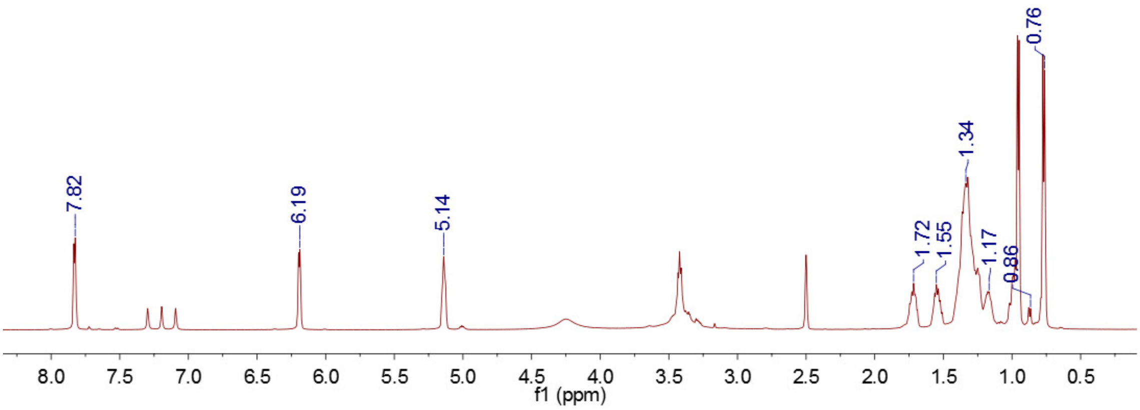

(**B**)

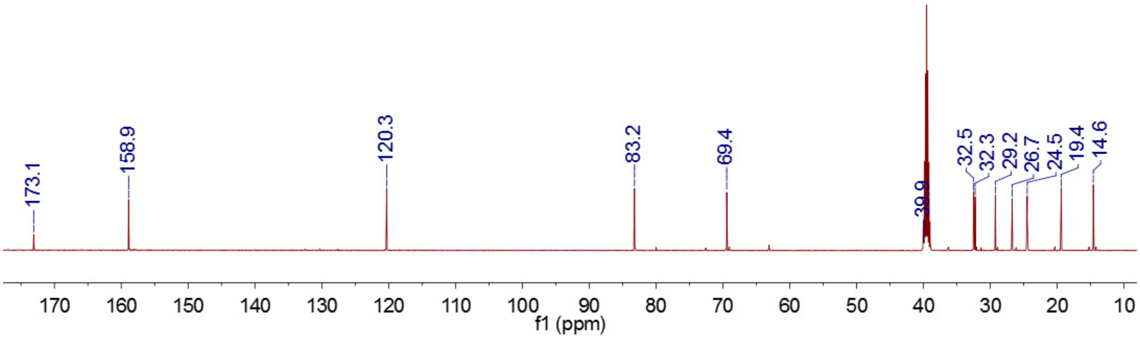

(**C**)

Figure S3. Spectral data of compound **3**; (**A**) The HR-ESIMS spectrum of compound **3**; (**B**) The ^{1}H-NMR spectrum of compound **3** in DMSO-d_6; (**C**) The ^{13}C-NMR spectrum of compound **3** in DMSO-d_6.

Table S1. ^{1}H and ^{13}C-NMR data of compound 2 and 3 in DMSO-d_6 (δ in ppm, *J* in Hz).

Position	2		3	
	δ_H	δ_C	δ_H	δ_C
1	-	173.1	-	173.1
2	6.19 (1H, d, 4.8)	120.3	6.19 (1H, d, 4.8)	120.3
3	7.84 (1H, d, 5.5)	158.9	7.82 (1H, d, 5.5)	158.9
4	5.15 (1H, m)	83.2	5.14 (1H, m)	83.2
5	1.71 (2H, m, H-5a)<br>1.56 (2H, m, H-5b)	32.5	1.72 (2H, m, H-5a)<br>1.55 (2H, m, H-5b)	32.5
6	1.27~1.35 (2H, m)	24.5	1.17~1.34 (2H, m)	24.5
7	1.27~1.35 (2H, m)	29.6	1.17~1.34 (2H, m)	29.2
8	1.27~1.35 (2H, m)	23.3	1.17~1.34 (2H, m)	26.7
9	1.27~1.35 (2H, m)	41.0	1.17~1.34 (2H, m)	32.3
10-OH	1.27~1.35 (m)	70.7	1.17~1.34 (1H, m)	39.9
11	1.27~1.35 (2H, m)	33.9	1.17~1.34 (1H, m)	69.4
12	0.79 (3H, t, 7.4)	8.4	0.86 (3H, d, 6.2)	19.4
13	0.98 (3H, s)	26.4	0.76 (3H, d, 6.6)	14.6
